# Olfactory dysfunction as a screening tool for mild and moderate cases of COVID-19: a single-center prevalence study of 646 patients in flu clinic

**DOI:** 10.1186/s43163-021-00186-7

**Published:** 2021-12-20

**Authors:** Anshika Harit, Pankaj Kumar, Ravi Prakash Jha

**Affiliations:** 1 Department of Otorhinolaryngology, Dr. Baba Saheb Ambedkar Medical College and Hospital, Rohini, Delhi, 110085 India; 2 Department of Community Medicine, Dr. Baba Saheb Ambedkar Medical College and Hospital, Rohini, Delhi, 110085 India

**Keywords:** Olfactory dysfunction, COVID-19, Anosmia, SARS-CoV-2, Smell dysfunction

## Abstract

**Background:**

To evaluate the prevalence of olfactory dysfunction (OD) in the Indian population and to establish olfactory dysfunction as a screening tool in COVID-19-positive patients. Data was collected using a questionnaire from laboratory-confirmed COVID-19 patients. The patient’s demographic and clinical details were analyzed to calculate the prevalence of olfactory dysfunction, general symptoms like fever, cough, malaise, diarrhea, along with the sinonasal symptoms. All the symptoms were self-reported, and no objective tests were carried out.

**Results:**

Out of 646 laboratory-confirmed cases of COVID-19 infection, olfactory dysfunction was self-reported by 465 (72%) patients and gustatory dysfunction (GD) was seen in 406 (62.8%) patients. The affected males (416) were proportionately more than females (230), with the mean age of our study population being 39.47 ± 13.85 (range 18–85 years). The most common symptoms were myalgia (*n* = 494, 76.5%), cough (*n* = 471, 72.9%), and fever (*n* = 444, 68.7%). Out of 465 patients with olfactory dysfunction, only 108 (23.2%) reported nasal obstruction. Five hundred thirty-three (82.5%) RT-PCR-positive patients did not give a history of smoking; however, co-morbidity was reported by 163 patients, of which 117 were found to have olfactory dysfunction. One hundred seventy (26.3%) patients gave a positive contact history.

13.6% reported olfactory dysfunction as their first symptom. A positive association was seen between olfactory dysfunction and gustatory dysfunction

**Conclusions:**

Our study demonstrates a high prevalence of 72% in the Indian population. We recommend that anosmia be used as a screening tool to identify mild to moderate cases of COVID-19.

## Background

December 2019 saw a significant portion of the Chinese population to get afflicted with lower respiratory tract illnesses. The causal agent identified as novel severe acute respiratory syndrome (SARS-CoV-2), was first reported from Wuhan, China [1]. WHO labeled COVID-19 (Coronavirus Disease-2019) as a global pandemic on 11 March 2020, as the infection rapidly spread across Asia, Europe, and America [2]. Variants of SARS-CoV were responsible for the Middle East respiratory syndrome in 2012–2013 (MERS-CoV) and SARS-CoV in 2002–2003 in China [3].

The initial presenting symptoms are fever, dry cough, dyspnea, and in severe cases, acute respiratory distress syndrome [1, 4]. Real-time reverse transcriptase-polymerase chain reaction (RT-PCR) technique and chest computed tomography (CT) are the standard diagnostic techniques [5, 6]. The other frequently reported manifestations of COVID-19 are anosmia/hyposmia, dysgeusia, diarrhea, headache, and myalgia [4]. The first notable Asian literature evaluating chemosensory dysfunction in COVID-19 patients did not report a substantial prevalence of hyposmia/anosmia in the Chinese population [7]. However, Lechein et al. reported a prevalence of 85.6% among their study population in their sizeable multicenter trial [8].

Our study aims to evaluate the prevalence of olfactory dysfunction (OD) in lab-confirmed cases of COVID-19 presenting to our hospital and establish OD as a screening tool to identify COVID-19 cases early.

## Methods

We conducted a single-center cross-sectional study research study in our hospital’s flu clinic after procuring approval from the institute’s ethics committee (F.5(50)/2020/BSAH/DNB/Committee/8218–8221). The flu clinic was made during the pandemic and all the patients who presented with fever and/or cough were directed to the clinic for initial evaluation and RT-PCR. Patients with respiratory distress were directed to the emergency for oxygen support and admission.

Only mild to moderate forms of coronavirus disease were considered for this study as it seemed ethically difficult to investigate olfactory and gustatory function in patients with severe life threatening infections.

Six thousand six hundred twenty-seven patients attended the clinic for screening from 15 April till 15 May 2021. Samples were collected using nasopharyngeal and throat swabs. Out of the total patients screened, 693 had positive real-time reverse transcriptase-polymerase chain reaction (RT-PCR) results.

Inclusion criteria entailed patients aged 18 and above, patients with mild to moderate disease which could independently fill out the questionnaire. We excluded patients with pre-existing smell disorders/taste disturbances, history of nasal surgery, head injury, psychiatric illness, chronic nasal disease, and severe cases requiring hospital admission or intensive care. On applying the inclusion criteria, our sample size came out to be 646. All the participants gave their informed written consent for the research study.

### Data collection

The patient filled out a questionnaire designed by the institution before the RT-PCR test. In the first section of the pro forma, the patients provided their demographic and epidemiological details like age/gender/address/identification proof. The second section dealt with the patient’s primary complaint, contact history, and any associated co-morbidities. The clinician filled out the patient’s vitals and the treatment advice at the end of the form. The patient’s symptoms were self-reported, and we did not conduct any objective tests to assess olfactory functions. Any information gap was covered by telephonic conversation. Subject experts analyzed the questionnaire to maximize the data output.

### Statistical methods

Descriptive statistics of the patient’s demographic characteristics and clinical features were calculated according to mean ± standard deviation (SD) in case of continuous variable, whereas frequency and percentage are provided in the case of a qualitative variable. A bar diagram was used to show the frequency of various self-reported symptoms. A clustered bar diagram was made to see the total number of positive COVID-19 cases among subgroups of gender and age group. Independent-sample *t* test was used for the comparison of the means. A chi-square test was applied to check the association between the categorical variables. A *p* value of less than 0.05 was considered statistically significant. All the Statistical analysis was performed using SPSS 26.0 (IBM, Armonk, New York).

## Results

**Fig. 1:**
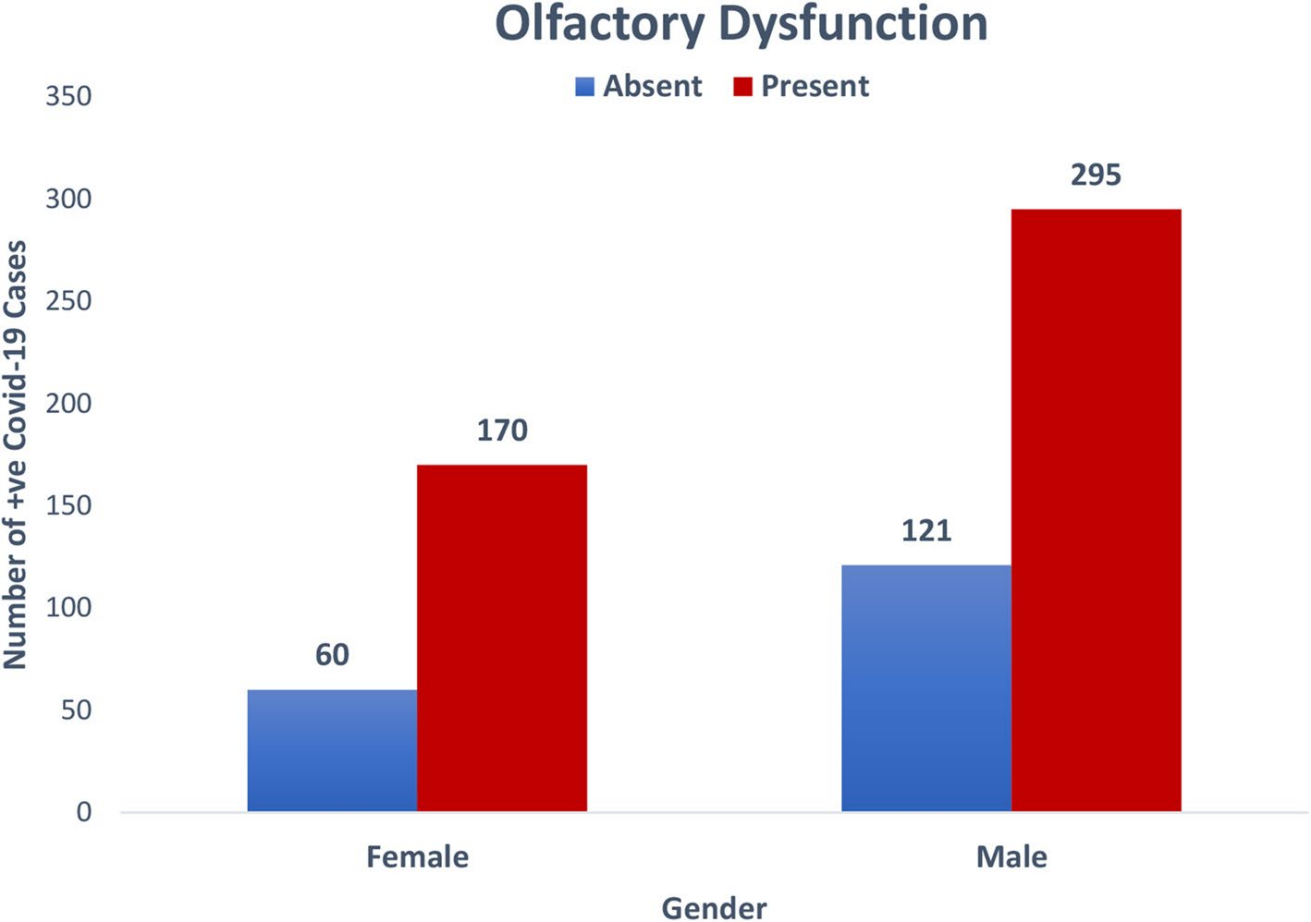
Gender distribution in COVID-19 patients

A total of 646 RT-PCR confirmed COVID-19 patients aged 18 and above were enrolled for the study. The authors took clearance from the institution’s ethical committee after a detailed presentation of the proposed research. Of that 646 laboratory-confirmed cases of COVID-19 infection, 416 were male, and 230 were female (Fig. 1). The mean age of our study population was 39.47 ± 13.85 (range 18–85 years) (Table 1). Majority of the patients were in 30–60 years age bracket.

**Table 1:** Demographic and clinical characteristics of COVID-19 patients

Variables	Frequency (%)/Mean ± S.D.
Gender	
Female	230 (35.6)
Male	416 (64.4)
Age	39.47 ± 13.85
18–30 years	187 (29.0)
30–60 years	395 (61.1)
60 years and above	64 (9.9)
Olfactory dysfunction	
Absent	181 (28.0)
Present	465 (72.0)
Gustatory dysfunction	
Absent	240 (37.2)
Present	406 (62.8)
Fever	
Absent	202 (31.3)
Present	444 (68.7)
Myalgia	
Absent	152 (23.5)
Present	494 (76.5)
Cough	
Absent	175 (27.1)
Present	471 (72.9)
Dyspnea	
Absent	372 (57.6)
Present	274 (42.4)
Headache	
Absent	278 (43.0)
Present	368 (57.0)
Sore throat	
Absent	313 (48.5)
Present	333 (51.5)
Nasal obstruction	
Absent	497 (57.6)
Present	149 (42.4)
Rhinorrhea	
Absent	410 (63.5)
Present	236 (36.5)
Diarrhea	
Absent	402 (62.2)
Present	244 (37.8)
Smoking	
Absent	533 (82.5)
Present	113 (17.5)
Co-morbidity	
Yes	163 (25.2)
No	483 (74.8)
Contact history	
Yes	170 (26.3)
No	476 (73.7)

**Fig. 2:**
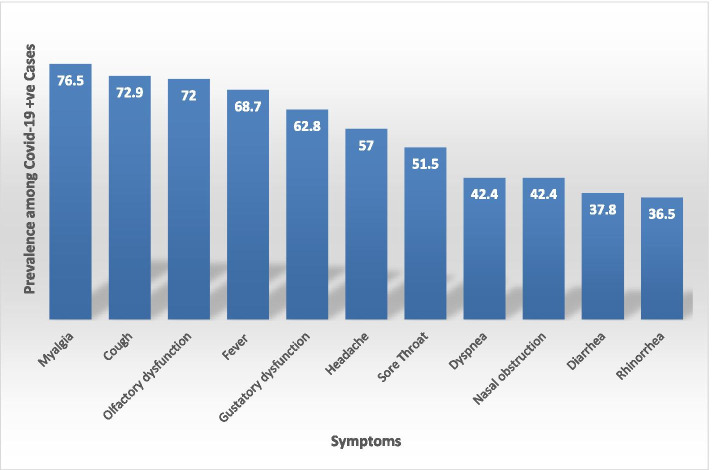
Bar chart depicting the prevalence of symptoms in COVID-19 patients

Olfactory dysfunction (OD) and gustatory dysfunction (GD) was self-reported by 465 (72%) and 406 (62.8%) patients respectively. Other frequent symptoms were myalgia (*n* = 494, 76.5%), cough (*n* = 471, 72.9%), fever (*n* = 444, 68.7%), headache (*n* = 368, 57%), dyspnea (*n* = 274, 42.4%), diarrhea (*n* = 244, 37.8%) (Table 1) (Fig. 2). Apart from OD, otolaryngological manifestations like sore throat (*n* = 333, 51.5%), rhinorrhea (*n* = 236, 36.5%) and nasal obstruction (*n* = 149, 42.4%) were also reported (Table 1).

However, out of 465 patients of OD, only 108 (23.2%) reported nasal obstruction. Moreover, rhinorrhea and sore throat was seen in 36.1% and 51% patients respectively (Table 2).

**Table 2:** Prevalence of sinonasal symptoms in COVID-19 patients with olfactory dysfunction

Variables	*N* (%)
Sore throat	
Absent	228 (49.0)
Present	237 (51.0)
Nasal obstruction	
Absent	357 (76.8)
Present	108 (23.2)
Rhinorrhea	
Absent	297 (63.9)
Present	168 (36.1)

In the present study, 533 (82.5%) PCR-positive patients did not report any history of smoking (Table 1). Out of which, 74 (65.5%) had associated smell disturbance. Underlying co-morbidity was reported by 163 patients of COVID-19 patients, of which 117 had OD (Table 3).

**Table 3:** Association of demographic variables with olfactory dysfunction

Variables	Without olfactory dysfunction (*n* = 181) Number (%)	With olfactory dysfunction (*n* = 465) number (%)	Total	*p* value
Mean age	39.75 ± 13.71	39.37 ± 13.91		0.293
Age groups				
18–30 years	49 (26.2)	138 (73.8)	187 (28.9)	0.719
30–60 years	112 (28.4)	283 (71.6)	395 (61.1)	
60 years and above	20 (31.3)	44 (68.8)	64 (9.9)	
Gender				
Female	60 (26.1)	170 (73.9)	230 (35.6)	0.416
Male	121 (29.1)	295 (70.9)	416 (64.4)	
Smoking				
Present	29 (25.7)	84 (74.3)	113 (17.5)	0.539
Absent	152 (28.5)	381 (71.5)	533 (82.5)	
Co-morbidity				
Yes	46 (28.2)	117 (71.8)	163 (25.2)	0.947
No	135 (28.0)	348 (72.0)	483 (74.8)	
Contact history				
Yes	47 (27.6)	123 (72.4)	170 (26.3)	0.900
No	134 (28.2)	342 (71.8)	476 (73.7)	

The most common underlying disease was hypertension, followed by diabetes, thyroid disease, and coronary artery disease (Table 4).

**Table 4:** Comorbidities seen in COVID-19 patients

Co-morbidity	*N* (%)
No	483 (74.8)
Hypertension	89 (13.8)
Diabetes	40 (6.2)
Thyroid	22 (3.4)
Coronary artery disease	9 (1.4)
Tuberculosis	7 (1.1)
Asthma	7 (1.1)
Chronic kidney disease	4 (0.6)
Autoimmune	5 (0.8)
COPD	4 (0.6)
Carcinoma	3 (0.5)

One hundred seventy (26.3%) patients gave a positive contact history (Table 1).

No significant association was present between olfactory loss/gustatory dysfunction and age, gender, smoking, or contact history (Table 2, Table 5).

**Table 5:** Association of demographic variables with gustatory dysfunction

Variables	Without gustatory dysfunction (*n* = 240) number (%)	With Gustatory dysfunction (*n* = 406) number (%)	Total	*p* value
Mean age	39.51 ± 13.48	39.45 ± 14.07		0.099
Age groups				
18–30 years	64 (34.2)	123 (65.8)	187 (28.9)	0.610
30–60 years	152 (38.5)	243 (61.5)	395 (61.1)	
60 years and above	24 (37.5)	40 (62.5)	64 (9.9)	
Gender				
Female	82 (35.7)	148 (64.3)	230 (35.6)	0.558
Male	158 (38.0)	258 (62.0)	416 (64.4)	
Smoking				
Present	39 (34.5)	74 (65.5)	113 (17.5)	0.523
Absent	201 (37.7)	332 (62.3)	533 (82.5)	
Co-morbidity				
Yes	67 (41.1)	96 (58.9)	163 (25.2)	0.227
No	173 (35.8)	310 (64.2)	483 (74.8)	
Contact history				
Yes	69 (40.6)	101 (59.4)	170 (26.3)	0.280
No	171 (35.9)	305 (64.1)	476 (73.7)	

Out of the 465 patients, 13.6% reported OD as their first symptom. 59.4% of the cases with hyposmia/anosmia had associated dysgeusia (Table 6).

**Table 6:** Correlation between olfactory dysfunction and gustatory dysfunction

Variables	Without gustatory dysfunction (*n* = 240) Number (%)	With gustatory dysfunction (*n* = 406) number (%)	Total	p- value
Olfactory dysfunction				
Yes	59 (40.6)	406 (59.4)	465 (72.0)	0.000
No	181 (35.9)	0 (0.0)	181 (28.0)	

## Discussion

Wuhan City in China reported the first known case of COVID-19 infection in December 2019 [9]. The disease has rapidly spread across continents and has already caused over a million deaths across the world (https://covid19.who.int/). Human-to-human transmission of the SARS-CoV-2 is possible via respiratory droplets, contact with an infected individual, or touching surfaces contaminated by SARS-CoV-2. The virus is detected using the RT-PCR technique. Samples collection is done using nasopharyngeal/oropharyngeal swabs [10].

COVID-19 majorly involves the lower respiratory tract and presents with a broad clinical spectrum ranging from no symptoms to fever, cough, dyspnea, which may rapidly progress to acute respiratory distress syndrome (ARDS) and death. Other frequently reported symptoms are malaise, myalgia/arthralgia, and diarrhea [11].

Mao et al. published one of the first reports of chemosensory dysfunction in COVID-19 patients from Asia. He reported hypogeusia in 5.6% and hyposmia in 5.1% cases. However, subsequent reports published in the European literature reported a higher prevalence of anosmia and dysgeusia [7].

A review article published in the initial phase of the pandemic by El-Anwar et al. compared the results of the various studies across the globe to see the common ENT manifestations in patients of coronavirus disease. In the analyzed results, fever and cough were dominant presentations. Among ENT manifestations, sore throat (11.3%) and headache (10.7%) were most commonly reported manifestation; however, olfactory dysfunction was sparsely described in the literature [12].

El-Anwar et al. also conducted a cross-sectional study on 120 patients of COVID-19 in which smell and taste dysfunction were seen in only 25% cases and the most common ENT manifestation were sore throat (30%) and nasal and congestion (28.3%). They also reported two unusual symptoms of tonsil enlargement and obstructive sleep apnea in 10% and 6.6% of their subjects respectively [13]. However, the most prominent ENT manifestations in our study were olfactory (72%) and gustatory dysfunction (62.8%), respectively. Also, none of our patients reported tonsil enlargement or obstructive sleep apnea.

A large retrospective multicenter European study conducted by Lechein reported olfactory dysfunction in 85.6% of patients and taste disturbances in 88% of cases. Four hundred seventeen lab-confirmed cases of COVID-19 participated in this study, out of which 11.8% of the patients reported olfactory dysfunction as their first symptom. Our results were comparable to the study conducted by Leichen et al., as 13.6% of cases reported OD as their primary symptom [8]. A research study conducted by Varia et al. used psycho-physiological objective tests of anosmia on 72 patients, out of which 18.1% of cases reported olfactory dysfunction as their first symptom [14].

Klopfenstein et al., in their research paper, retrospectively observed features of anosmia in 114 patients. 47% of the patients reported anosmia. Fatigue (93%), cough (87%), fever (74%), headache (82%), myalgia (74%), and diarrhea (52%) were predominant symptoms. Our study results were in line with Klopfenstein’s findings, with myalgia (76.5%), cough (72.9%), and fever (68.7%) being the most typical systemic manifestations. Also, OD was associated with GD in 59.4% of cases [15].

Another similarity to this study was the frequency of sinonasal symptoms like nasal obstruction and rhinorrhea in patients with OD. Klopfenstein observed rhinorrhea in 57% and nasal obstruction in 30% cases of OD. While in our study, the prevalence of rhinorrhea and nasal obstruction were 36.1% and 23.2%, respectively.

However, unlike many studies, our study had proportionately more male patients as compared to female. This result was comparable to the study carried out by Al Ani et al., who observed that males were affected more than females. The prevalence of smokers was similar to our study [16].

Spinato et al. used the SNOT 22 grade to categorize the OD/GD into mild-moderate and severe. 64.4 and 57.3% of patients reported disturbances in taste and smell, respectively [17].

Our results were in line with the findings of Luers et al.; the affected males were proportionately more than females. Out of the 72 patients enrolled for the study, 53 had anosmia (73.6%), and 69.4 had GD [18].

Moein et al. reported the highest incidence of OD in all the studies reviewed. Ninety-five percent of patients reported OD [19].

On comparing our study with our Indian counterparts, Mishra et al. conducted a case-control trial with 74 patients in each group; we observed that the result of gender predisposition was similar to our observation. However, the prevalence of anosmia in their research was only 14.8% [20].

Post viral smell dysfunction is not uncommon and has been reported with the respiratory syncytial virus, influenza, and parainfluenza viruses [21]. It may be due to the olfactory cleft area’s congestion and edema, secondary inflammation of the nasal mucosa, or neurological deficit due to olfactory bulb damage [22]. However, the high prevalence of anosmia associated with COVID-19 needs further in-depth evaluation. Our study results in terms of the prevalence of OD and GD are comparable to our European counterparts.

## Conclusions

Olfactory dysfunction can be the first symptom of COVID-19 and may precede the development of a more severe infection. Considering that three fourth of our study population had an alteration of smell and taste, we recommend that OD be an independent and primary criterion for suspected COVID-19 patients. Further, in-depth trials utilizing objective tests should are needed for further evaluation and characterization COVID-19.

## Data Availability

Patient’s personal details have not been made available due to privacy concerns. However, the raw data collected from the flu clinic is available for analysis.

## References

[1] Huang C, Wang Y, Li X, Ren L, Zhao J, Hu Y (2020). Clinical features of patients infected with 2019 novel coronavirus in Wuhan. China Lancet.

[2] Cucinotta D, Vanelli M (2020). WHO declares COVID-19 a pandemic. Acta Biomed.

[3] Wit de E, Doremalen van N, Falzarano D, Munster VJ. SARS and MERS: recent insights into emerging coronaviruses. Nat Rev Microbiol 2016; 14(8): 523–534 10.1038/nrmicro.2016.81 PMC7097822 27344959

[4] Rodriguez-Morales AJ, Cardona-Ospina JA, Gutiérrez-Ocampo E, Vil- lamizar-Peña R, Holguin-Rivera Y, Escalera-Antezana JP, et al. Clinical, laboratory and imaging features of COVID-19: a systematic review and meta-analysis. Travel Med Infect Dis. 2020 Mar; 34:101623 10.1016/j.tmaid.2020.101623 PMC7102608 32179124

[5] Singhal T (2020). A review of coronavirus disease-2019 (COVID-19). Indian J Pediatr.

[6] Ai T, Yang Z, Hou H, Zhan C, Chen C, Lv W, et al. Correlation of chest CT and RT-PCR testing for coronavirus disease 2019 (COVID-19) in China: A report of 1014 Cases. Radiology. (2020). Available from: 10.1148/radiol.2020200642 10.1148/radiol.2020200642 PMC7233399 32101510

[7] Mao L, Jin H, Wang M, Hu Y, Chen S, He Q (2020). Neurological manifestations of hospitalized patients with coronavirus disease 2019 in Wuhan, China: a retrospective case series study. JAMA Neurol.

[8] Lechien JR, Chiesa-Estomba CM, De Siati DR, Horoi M, Le Bon SD, Rodriguez A, et al. Olfactory and gustatory dysfunctions as a clinical presentation of mild-to-moderate forms of the coronavirus disease (COVID-19): a multicentre European study. Eur Arch OtoRhinoLaryngol 2020. Available from: 10.1007/s00405-020-05965-1 10.1007/s00405-020-05965-1 PMC7134551 32253535

[9] Wang D, Yin Y, Hu C, Liu X, Zhang X, Zhou S (2020). Clinical course and outcome of 107 patients infected with the novel coronavirus, SARS-CoV-2, discharged from two hospitals in Wuhan. China Critical care.

[10] He JL, Luo L, Luo ZD, Lyu JX, Ng MY, Shen XP (2020). Diagnostic performance between CT and initial real-time RT-PCR for clinically suspected 2019 coronavirus disease (COVID-19) patients outside Wuhan. China Respir Med.

[11] Menni C, Valdes A, Freydin MB, Ganesh S, Moustafa JE, Visconti A, et al. Loss of smell and taste in combination with other symptoms is a strong predictor of COVID-19 infection. Nat Med. 2020. Available from: doi: 10.1038/s41591-020-0916-2

[12] El-Anwar MW, Elzayat S, Fouad YA (2020). ENT manifestation in COVID-19 patients. Auris Nasus Larynx.

[13] El-Anwar, Mohammad Waheed et al. Analysis of ear, nose and throat manifestations in COVID-19 patients. Int. Arch. Otorhinolaryngol [online]. 2021, v. 25, n. 3 [Accessed 16 October 2021] , pp. 343–348. Available from: <10.1055/s-0041-1730456> 10.1055/s-0041-1730456 PMC8321632 34377166

[14] Vaira LA, Deiana G, Fois AG, Pirina P, Madeddu G, Vito De A, et al. Objective evaluation of anosmia and ageusia in COVID-19 patients: single-center experience on 72 cases. Head Neck 2020; 42:1252–1258 10.1002/hed.26204 PMC7267244 32342566

[15] Klopfenstein T, Kadiane-Oussou NJ, Toko L, Royer PY, Lepiller Q, Gendrin V (2020). Features of anosmia in COVID-19. Med Mal Infect.

[16] Al-Ani RM, Acharya D (2020). Prevalence of anosmia and Ageusia in patients with COVID-19 at a primary health center, Doha, Qatar. Indian J Otolaryngol Head Neck Surg..

[17] Spinato G, Fabbris C, Polesel J, Cazzador D, Borsetto D, Hopkins C (2020). Alterations in smell or taste in mildly symptomatic outpatients with SARS-CoV-2 infection. JAMA..

[18] Luers JC, Klussmann JP, Guntinas-Lichius O (2020). The COVID-19 pandemic and otolaryngology: what it comes down to?. Laryngorhinootologie..

[19] Moein ST, Hashemian SMR, Mansourafshar B, Khorram-Tousi A, Tabarsi P, Doty RL (2020). Smell dysfunction: a biomarker for COVID-19. Int Forum Allergy Rhinol.

[20] Mishra P, Gowda V, Dixit S, Kaushik M (2020). Prevalence of new-onset anosmia in COVID-19 patients: is the trend different between European and Indian population?. Indian J Otolaryngol Head Neck Surg.

[21] Suzuki M, Saito K, Min W-P, Vladau C, Toida K, Itoh H (2007). Identification of viruses in patients with post-viral olfactory dysfunction. Laryngoscope..

[22] Naeini AS, Karimi-Galougahi M, Raad N, Ghorbani J, Taraghi A, Haseli S (2020). Paranasal sinuses computed tomography findings in anosmia of COVID-19. Am J Otolaryngol.

